# Corticosterone binds α_1A_-adrenoceptors with high affinity and induces strain-dependent vascular desensitization in female spontaneously hypertensive rats

**DOI:** 10.1038/s41440-026-02760-z

**Published:** 2026-08-06

**Authors:** Yanina A. Santander-Plantamura, Karen Nuñez-Solano, Santiago C. Sigrist-Flores, Itzell A. Gallardo-Ortíz, Enrique Hong, Rafael Villalobos-Molina

**Affiliations:** 1 Unidad de Investigación en Biomedicina, Facultad de Estudios Superiores Iztacala, Universidad Nacional Autónoma de México, Tlalnepantla, México; 2 Carrera de Químico Farmacéutico Biólogo, Facultad de Estudios Superiores Zaragoza, Universidad Nacional Autónoma de México, Iztapalapa, México; 3 Carrera de Médico Cirujano, Facultad de Estudios Superiores Iztacala, Universidad Nacional Autónoma de México, Tlalnepantla, México; 4 Departamento de Farmacobiología, Centro de Investigación y de Estudios Avanzados, Sede Sur, México

**Keywords:** Corticosterone, α1A-adrenoceptor, Vascular reactivity, Molecular docking, Morning hypertension, Digital hypertension

## Abstract

Glucocorticoids are classically associated with hypertension, but their acute vascular effects remain incompletely understood. Female rats were selected due to their higher basal and stress-induced corticosterone levels, making them a physiologically relevant model. We investigated whether corticosterone directly modulates α1-adrenoceptor-mediated vasoconstriction in a strain- and age-dependent manner in female spontaneously hypertensive (SHR) and normotensive Wistar Kyoto (WKY) rats. Vascular reactivity was evaluated in pithed rats and isolated aortic rings from SHR and WKY rats across different ages (young, aged, senescent). Concentration-response curves to phenylephrine were performed under control conditions, after acute corticosterone, and in the presence of selective α_1A_-AR (5-methylurapidil) or α_1D_-AR (BMY-7378) antagonists. Molecular docking assessed corticosterone binding affinity to α1-adrenoceptor subtypes. In pithed rats, corticosterone decreased phenylephrine sensitivity (increased ED_50_) in young and aged SHR, with no effect in WKY. In aortic rings, corticosterone reduced phenylephrine sensitivity (decreased pD₂) exclusively in SHR. Principal component analysis revealed that corticosterone-treated SHR curves clustered with α_1A_-AR antagonist treatment, suggesting shared signaling. Deviation from additivity analysis (%ΔAUC) showed antagonism between corticosterone and 5-methylurapidil in SHR. Docking demonstrated high-affinity corticosterone binding to α_1A_-AR (ΔG = -9.14 kcal/mol; Ki = 198.13 nM). Pharmacological and docking evidence strongly suggest that corticosterone selectively desensitizes α1-adrenoceptor-mediated vasoconstriction in SHR through high-affinity α_1A_-AR binding, although alternative mechanisms may contribute. This effect is preserved in young and aged SHR, but lost in senescent animals. This strain-specific, age-dependent interaction reveals a novel non-genomic glucocorticoid action on the vasculature, with implications for blood pressure regulation in hypertension and aging.

**Figure:**
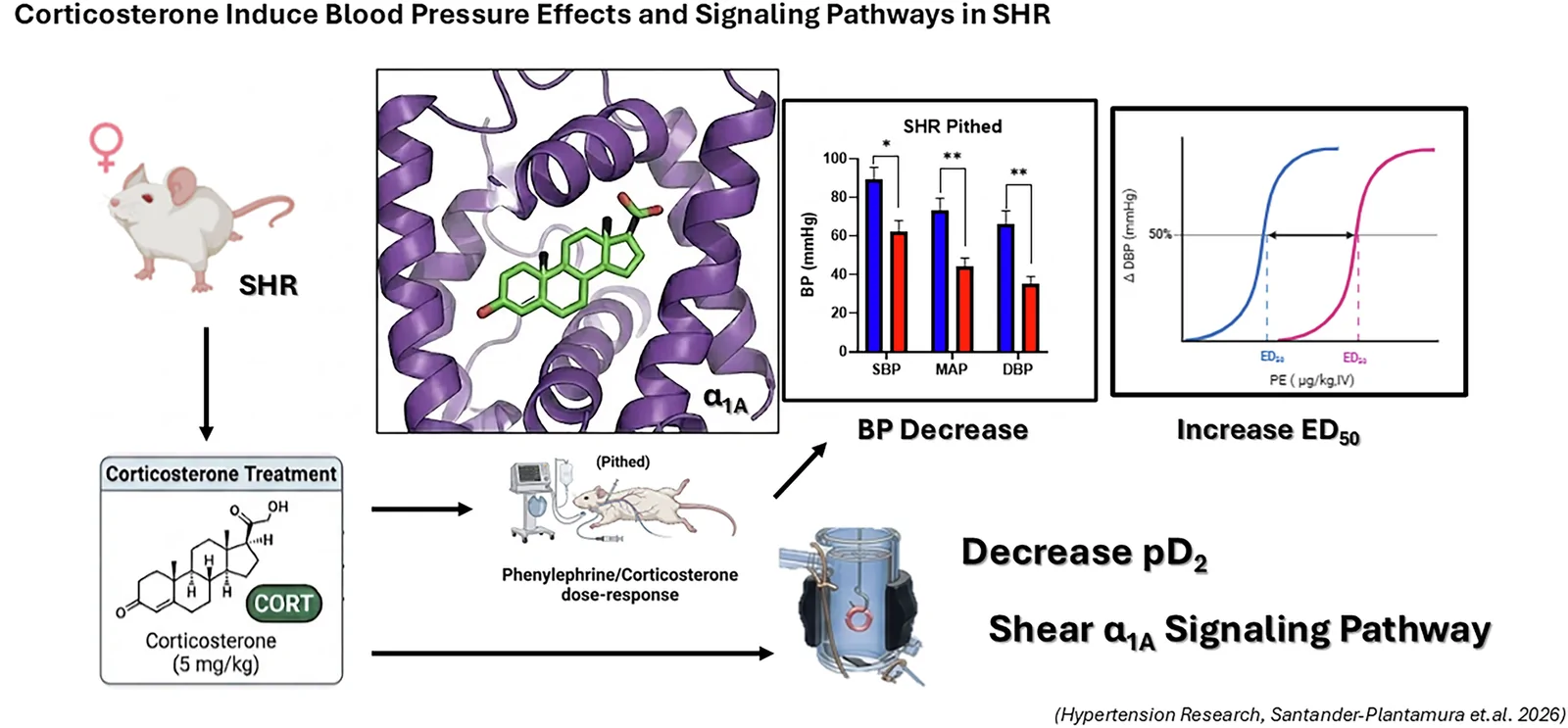

## Introduction

Hypertension, defined as the persistent elevation of systolic and/or diastolic blood pressure, remains a leading modifiable risk factor for cardiovascular morbidity and mortality worldwide [1]. Essential or primary hypertension, which accounts for 90–95% of all hypertensive cases, is a multifactorial disorder influenced by genetic, environmental, and social factors, with no clearly identifiable underlying cause [2,3]. Key pathogenic mechanisms include increased sympathetic nervous system activity, dysregulation of the renin-angiotensin-aldosterone system (RAAS), sodium retention, an imbalance between vasodilatory and vasoconstrictor pathways, and oxidative stress-mediated endothelial dysfunction [4].

Adrenergic receptors, which mediate the physiological actions of catecholamines, are central to cardiovascular regulation. Among them, the α_1_-adrenoceptor family (α_1A_, α_1B_, and α_1D_) plays distinct roles in the control of vascular tone. The α_1D_-adrenoceptor is highly expressed in conduit arteries and has been implicated in pathological vasoconstriction, vascular remodeling, and the maintenance of elevated blood pressure (BP) in hypertensive models [5–9]. Notably, a population of constitutively active α_1D_-adrenoceptors has been identified in the arteries of male SHR, linking this receptor subtype directly to the pathophysiology of hypertension [7,10]. Furthermore, pharmacological blockade of α_1D_-adrenoceptors with BMY-7378 reduces vascular hypertrophy in Ang II-induced hypertension, reinforcing its role in vascular remodeling [11].

Neuroendocrine mechanisms, including glucocorticoid excess, also contribute to the development of hypertension. Elevated circulating corticosterone has been reported in SHR compared to normotensive Wistar Kyoto (WKY) rats, accompanied by an exaggerated pressor response to stress [12]. Interestingly, the α_1A_-adrenoceptor has emerged as a key modulator of vascular tone through a distinct mechanism. Arce et al. [13] demonstrated that activation of α_1A_-adrenoceptors in the rat aorta triggers a desensitization process, mediated by endothelial neuronal nitric oxide synthase (nNOS), which attenuates vasoconstriction induced by high concentrations of α_1_-AR agonists. This α_1A_/nNOS pathway acts as a physiological “brake” against excessive adrenergic stimulation, but it is lost with ageing, paralleling the decline in α_1A_-adrenoceptor expression [13]. This mechanism may represent a critical target for glucocorticoid modulation in hypertension.

Glucocorticoids are known to potentiate Ang II signaling in the vasculature [14], and to modulate α_1_-adrenoceptors. For instance, corticosterone induces receptor desensitization in the brain [15] and regulates α_1D_-adrenoceptor expression [16]. Importantly, sex differences exist in glucocorticoid concentrations: female rats exhibit higher basal and stress-induced corticosterone concentrations than males, a difference influenced by estrogen-dependent modulation [17].

Despite this background, the functional interaction between corticosterone and α_1_-adrenoceptors in the vasculature, particularly in the context of ageing and hypertension, remains poorly understood. Given that female rats exhibit higher basal and stress-induced corticosterone than males, influenced by estrogen-dependent modulation, we selected female SHR and WKY rats for this study to investigate glucocorticoid-vascular interactions in a physiologically relevant context. The aim of this study was to determine whether corticosterone directly modulates α_1_-adrenoceptor-mediated vascular reactivity in female hypertensive and normotensive rats, and whether this effect is modified by age and the hypertensive phenotype. We hypothesized that corticosterone contributes to BP regulation through differential modulation of α_1_-adrenoceptors, and that these effects are influenced by age and the hypertensive phenotype.

## Materials and methods

### Drugs preparation

Corticosterone (Sigma-Aldrich, Cat. No. 27840) was freshly dissolved on the day of the experiment in a vehicle, consisting of 10% ethanol and 90% isotonic saline (0.9% NaCl) to a final concentration of 2 mg/mL, as described [18]. This dose was selected based on previous studies demonstrating that intraperitoneal administration of corticosterone at 5 mg/kg produces rapid behavioral effects within minutes of administration, and that this dose produces plasma concentrations mimicking those observed during substantial stress [18].

BMY-7378, an α_1D_-adrenoceptor antagonist (Sigma-Aldrich, CAS. 21102-95-4), was prepared at 1 mg/mL in sterile saline (0.9% NaCl) and administered intravenously (i.v.) at 1 mg/kg. For isolated aortic assay, 5-Methylurapidil, an α_1A_-adrenoceptor antagonist (Research Biochemichals International, CAS. 34661-75-1).

### Animals

A total of 60 female rats were used in this study, with 30 rats per strain (SHR and WKY). For each strain, rats were divided into three age groups (young, aged, senescent; *n* = 10 per age group), and further subdivided into pithed (*n* = 5) and conscious (*n* = 5) preparations for blood pressure measurements and vascular reactivity studies. For isolated organ bath experiments, aortic rings from 3–5 rats per group were used, consistent with standard practice in vascular pharmacology. All rats were in proestrous phase confirmed by vaginal cytology [19]. All procedures were approved by the Institutional Animal Care and Use Committee (Protocol No. 1497) and adhered to national (NOM-062-ZOO-1999, SAGARPA, Mexico), and international (NIH Guide for the Care and Use of Laboratory Animals) guidelines. Animals were housed under controlled conditions (22 ± 2°C, 40–60% humidity, 12:12 h light/dark cycle) with *ad libitum* access to food and water.

### Experimental protocol

Female SHR and WKY rats were divided into three age groups: young (3 months), aged (6 months), and senescent (12 months). Tail-cuff blood pressure (BP) was measured before and after a single intraperitoneal injection of corticosterone (5 mg/kg) or vehicle. Estrous cycle phase (proestrus) was confirmed by vaginal cytology before BP recording and serum collection. A separate pithed cohort received intravenous phenylephrine with or without corticosterone pretreatment (5 mg/kg, i.p., 30 min before). Thoracic aortas were collected for isolated organ bath studies.

### Corticosterone serum by ELISA

Corticosterone concentrations were measured by a competitive enzyme immunoassay (Corticosterone ELISA Kit, EIACORT; Thermo Fisher Scientific), following the manufacturer’s protocol. Serum samples were treated with Dissociation Reagent and diluted 1:100 prior to analysis. Absorbance was measured at 450 nm using an Epoch microplate spectrophotometer (BioTek Instruments, Winooski, VT, USA), and sample concentrations were interpolated from a four-parameter logistic standard curve. All blood samples were collected by retro-orbital puncture under anesthesia with isoflurane, between 8 and 9 am [20] in proestrus phase. Corticosterone were measured only under basal conditions (i.e., prior to any exogenous corticosterone administration), as the aim was to characterize strain- and age-dependent differences in endogenous glucocorticoid concentrations.

### Pithed rats and dose–response curves to phenylephrine

Pithed rat preparation was performed as previously described [21]. Rats were anesthetized with sodium pentobarbital (70 mg/kg, i.p.), pithed, and ventilated with room air. Bilateral vagotomy was performed. Catheters were placed in the left femoral vein for drug delivery and in the left carotid artery for blood pressure recording. After stabilization, cumulative doses of phenylephrine (PE; 0.1–310 µg/kg, i.v.) were administered. Each dose was injected at a volume of body weight/10 µL followed by saline flush, and the next dose was given only after the pressor response returned to baseline [22]. Diastolic blood pressure (DBP) responses were recorded, and vasoconstriction was quantified as ΔDBP (mmHg). BMY-7378 (1 mg/kg, i.v.) or corticosterone (5 mg/kg, i.p.) was administered 15 or 30 min before PE, respectively. Controls received vehicle.

### Isolated organ bath studies

Thoracic aortas were dissected, cleaned, and cut into 3 mm rings. Rings were mounted in organ baths containing Krebs-Henseleit solution at 37°C, pH 7.4, bubbled with 95% O₂/5% CO₂. Isometric tension was recorded under a resting tension of 3 g after 60 min equilibration [23]. Cumulative concentration-response curves to phenylephrine (PE; 1×10⁻⁹ M to 1×10⁻⁴ M) were constructed. Endothelial integrity was assessed by acetylcholine-induced relaxation (10⁻⁹ M to 10⁻⁵ M); rings with <70% (WKY) or <35% (SHR) relaxation were excluded. To evaluate pharmacological modulation, rings were pre-incubated (30 min) with corticosterone (10⁻⁶ M), BMY-7378 (10⁻⁷ M, α_1D-_AR antagonist), 5-methylurapidil (5MU, 10⁻⁷ M, α_1A-_AR antagonist), or their combinations. Experiments were performed in the presence of yohimbine and propranolol (both 10⁻⁷ M) to block α₂- and β-adrenoceptors. KCl (80 mM) served as a receptor-independent control.

### Tail-cuff blood pressure measurement basal and post corticosterone i.p. administration

Non-pithed rats were trained by restrainer at 37°C for 1 week. Tail blood pressure was measured using plethysmography (IITC Life Science, MRBP, CA, USA), and three readings per rat were averaged to obtain BP. To evaluate systemic effect, corticosterone (5 mg/kg, i.p.) was administered to non-pithed rats. After administration, a 30-min stabilization period was allowed to ensure proper distribution and physiological action of the hormone. BP was measured using the tail-cuff method. In pithed rats, we evaluated the effect of corticosterone, at the same dose, by carotid artery cannulation.

### Statistical analysis

The sample sizes used (*n* = 3–5 per group for isolated organ bath experiments, *n* = 5 per group for pithed rat experiments) are consistent with standard practice in vascular pharmacology and are sufficient to detect biologically significant differences in pharmacodynamic parameters (pD₂, E_max_) with an expected effect size of approximately 0.5–0.8 standard deviations. However, we acknowledge that the relatively small sample sizes limit statistical power for subgroup comparisons, particularly in the multivariate PCA and correlation analyses. While PCA is a data reduction technique that can be applied to relatively small datasets, the stability of the loadings and the generalizability of the results should be interpreted with caution. Similarly, the correlation analysis provides preliminary insights but should be confirmed in larger cohorts. Individual data points are included in all figures to allow readers to assess the variability and distribution of the data.

Data normality was assessed with the Shapiro-Wilk test (*n* ≤ 50). Parametric tests were analyzed using paired Student’s *t*-test, or one-way ANOVA followed by Tukey’s *post hoc* test. Non-parametric data were evaluated with the Wilcoxon matched-pairs test or Kruskal-Wallis test, followed by Dunn’s *post hoc* correction. Analyses were conducted using GraphPad Prism 9.0 (RRID:SCR_002798). Results are expressed as mean ± SEM (parametric) with *p* < 0.05 considered statistically significant.

Dose-response curves were fitted using nonlinear regression (log[agonist] *vs*. response, variable slope, four-parameter model), and ED_50_ values compared by one-way ANOVA and Tukey’s *post hoc* test. All results are presented as mean ± SEM (*p* < 0.05, considered significant).

Multivariate analysis was performed using Principal Component Analysis (PCA) to integrate pharmacodynamic parameters (pD_2_, E_max_, KCl, and Relative E_max_) into a single functional space. Data preprocessing and scaling were executed using a custom Python script (Supplementary Data 2). Prior to analysis, all variables were standardized using a Z-score transformation (StandardScaler), to ensure that differences in units did not bias the PCA calculation.

The PCA was implemented using the Scikit-learn library. The first two principal components (PC1 and PC2) were selected for visualization as they accounted for most of the total variance. Factor loadings were calculated to determine the contribution of each physiological parameter to the spatial distribution of the experimental cohorts [24].

The interaction was evaluated using a fixed-dose-response additivity protocol. The theoretical additive effect was calculated as the sum of the individual inhibitory effects of Corticosterone, and the respective adrenergic blocker (BMY-7378 or 5-MU) on the Phenylephrine-induced contraction curve. Deviations from this theoretical sum were expressed as ∆AUC% [25].

### Data visualization

Graphical representation was generated using a multi-panel biplot layout. Data points were categorized by strain (WKY and SHR), age group (Young, Aged, and Senescent), and pharmacological treatment (PE, BMY, 5MU, and CORT). Visualization was performed using the Seaborn and Matplotlib libraries, employing distinct markers and color palettes to enhance the discriminative power between groups. The final figures represent the spatial convergence or divergence of vascular profiles, with red vectors indicating the direction and magnitude of the underlying loading variables. To further explore the relationships between systemic, endocrine, and vascular parameters, a heat map was constructed based on Spearman’s rank correlation matrix. For this analysis, data from all experimental groups were pooled, and pairwise correlation coefficients were calculated using the corr() function from the Pandas library, followed by hierarchical clustering using the Seaborn clustering function. The heat map was visualized using a diverging color palette (coolwarm), where red tones represent positive correlations, blue tones represent negative correlations, and color intensity reflects the strength of the correlation coefficient (r).

### Molecular docking

The crystal structure of the α_1A_-adrenoceptor was obtained from the Protein Data Bank (PDB ID: 7YMH) [26]. The α_1D_-adrenoceptor structure was generated by homology modeling using the Robetta server [27], based on the α_1A_ template (7YMH). Ligand and receptor preparation were performed using UCSF Chimera [28]. Docking was conducted using AutoDock Vina [29] and AutoDock4 [30]. Grid boxes (40×40×40 Å, 0.375 Å spacing) were centered at x = 118.858, y = 121.960, z = 143.227 for α_1A_, and at x = 129.636, y = 121.940, z = 139.103 for α_1D_. The protocol was validated by redocking the co-crystallized ligand into 7YMH (RMSD < 2.0 Å). Binding energies (ΔG), inhibition constants (Ki), and interaction profiles were analyzed using Discovery Studio 4.0. Model quality was assessed with MolProbity [31,32] and ERRAT [33]; see Supplementary Data 3.

## Results

### Blood pressure evaluation

SHR rats showed higher BP than WKY rats from as early as 3 months of age (Supplementary data 1, Table 1), in agreement with previous reports [12]. Acute corticosterone administration (5 mg/kg, i.p.) did not alter blood pressure in conscious rats of any group (data not shown).

**Fig. 1:**
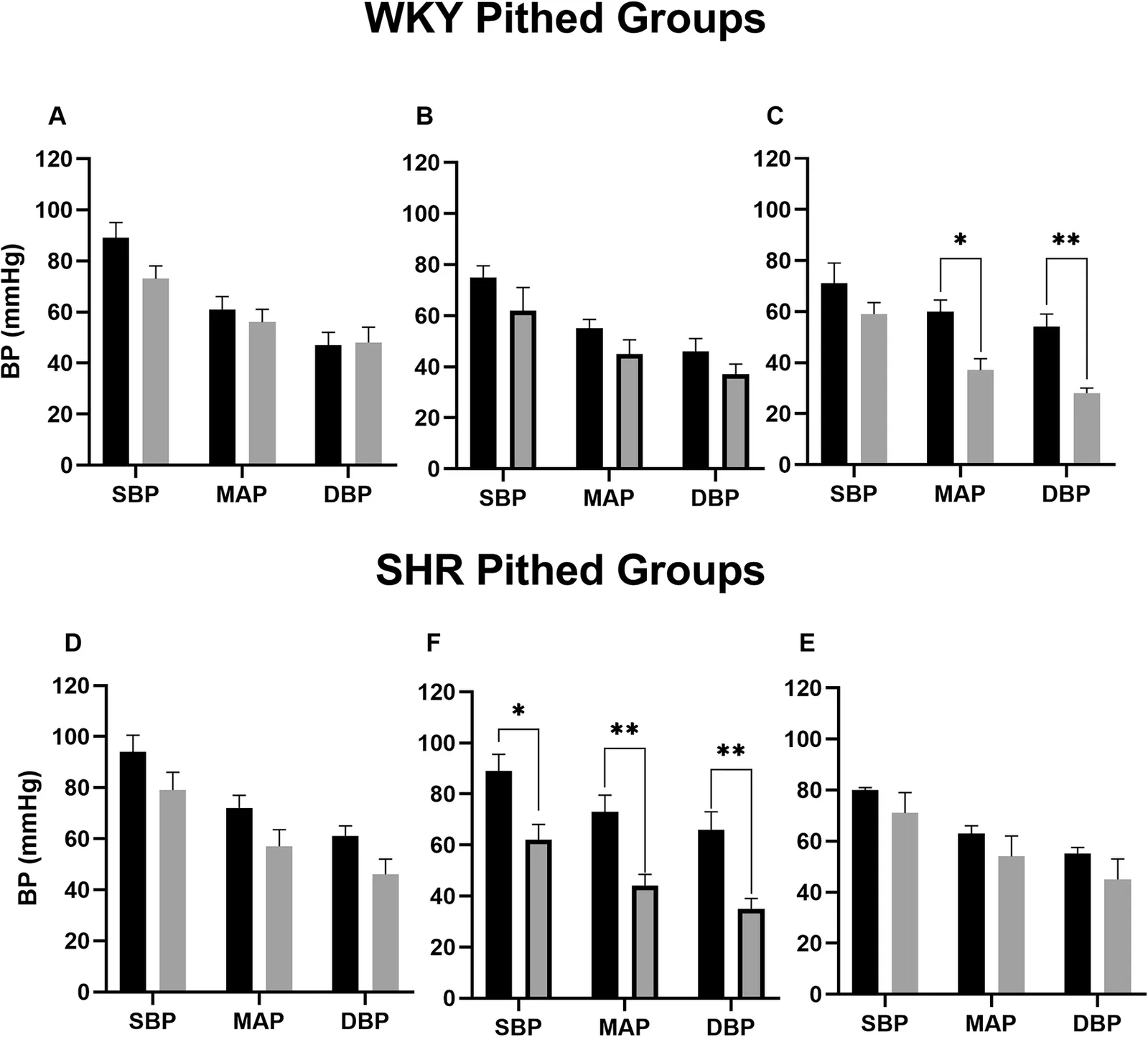
Effect of acute corticosterone administration on systolic, mean, and diastolic blood pressure in pithed rats. Systolic (SBP), mean (MAP), and diastolic (DBP) blood pressure were recorded in pithed female rats 30 min after acute administration of corticosterone (5 mg/kg, i.p.) or vehicle. Experiments were performed in young **A, D**, Aged **B, E** and Senescent **C, F** rats. Data are expressed as mean ± SEM (*n* = 5 per group). *p < 0.05, **p < 0.01 *vs*. respective vehicle control (unpaired *t*-test). SHR spontaneously hypertensive rats, WKY Wistar Kyoto rats

In pithed rats, acute administration of corticosterone (5 mg/kg, i.p.) induced a significant hypotensive effect in aged SHR rats and, notably, in senescent WKY rats (Fig. 1). No significant changes in blood pressure were observed in young or aged WKY rats, or in young or senescent SHR rats following corticosterone administration.

### Systemic vascular responsiveness

**Fig. 2:**
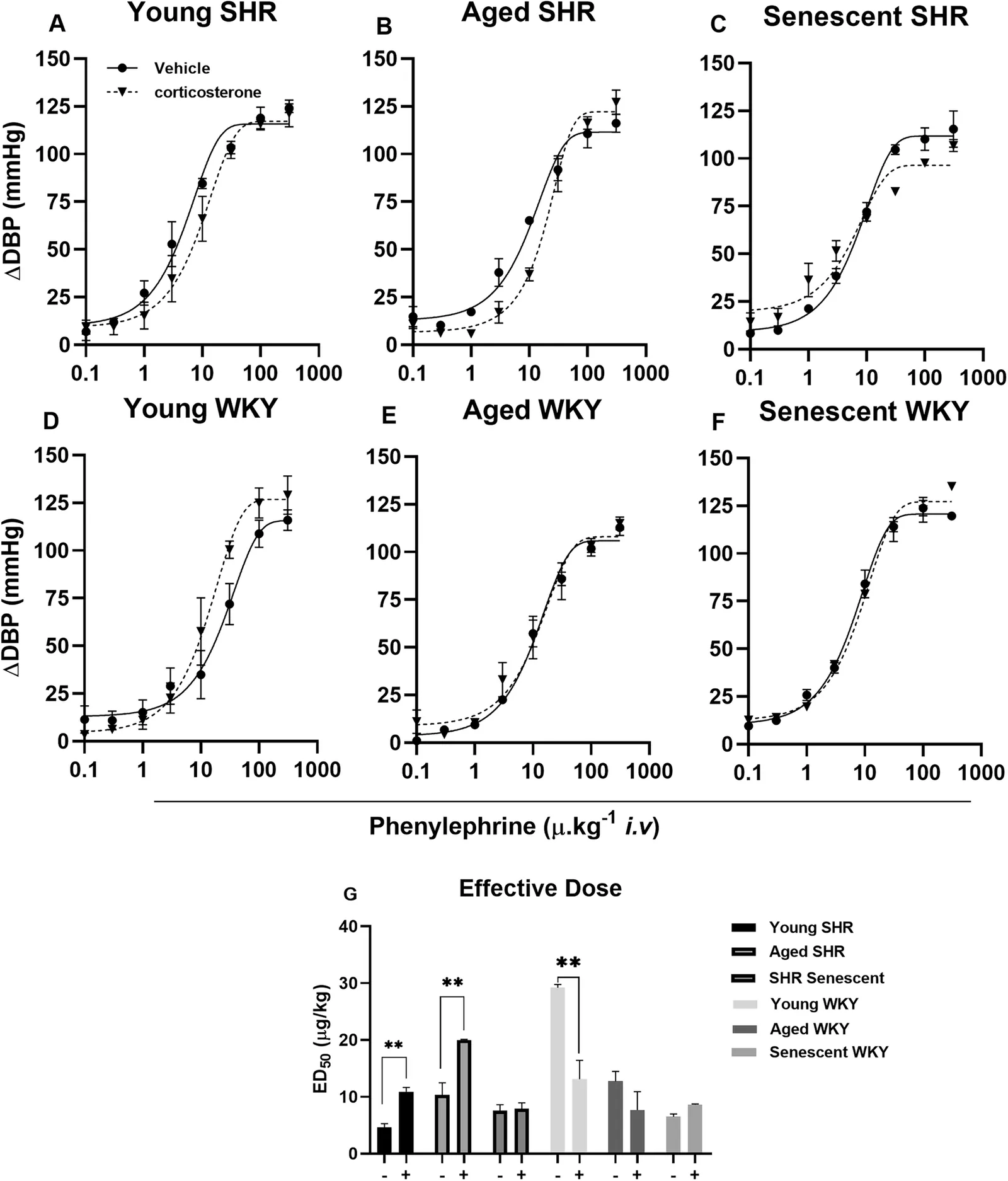
Strain- and age-dependent modulation of phenylephrine dose-response curves and ED_50_ by corticosterone in pithed rats. Phenylephrine dose-response curves under control conditions (vehicle -) and after acute corticosterone (+, 5 mg/kg, i.p.) in pithed female rats: **A** young SHR, **B** aged SHR, **C** senescent SHR, **D** young WKY, **E** aged WKY, and **F** senescent WKY. Data are mean ± SEM (*n* = 6–8). **G** Corresponding ED_50_ values. **p < 0.01 *vs*. vehicle

Peripheral vascular reactivity was assessed as the change in diastolic blood pressure (ΔDBP) from baseline, since DBP primarily reflects peripheral vascular resistance and is therefore the most appropriate parameter to evaluate α_1_-adrenoceptor-mediated vasoconstriction in pithed rats [34]. In pithed rats, acute corticosterone administration (5 mg/kg, i.p.) significantly decreased PE sensitivity (increased ED_50_) in both young and aged SHR rats, indicating a desensitizing effect of the glucocorticoid on α_1_-adrenoceptor-mediated vasoconstriction in the hypertensive strain (Fig. 2). Interestingly, in young WKY rats, corticosterone produced the opposite effect, increasing PE sensitivity (decreased ED_50_). No significant effect of corticosterone was observed in aged or senescent WKY rats (Fig. 2). No significant changes in Emax were observed across groups, indicating that the effects were primarily on sensitivity rather than in maximal efficacy. All of the pharmacodynamic parameters information is in Supplementary Data 1, Table 2 and Fig. 1.

**Fig. 3:**
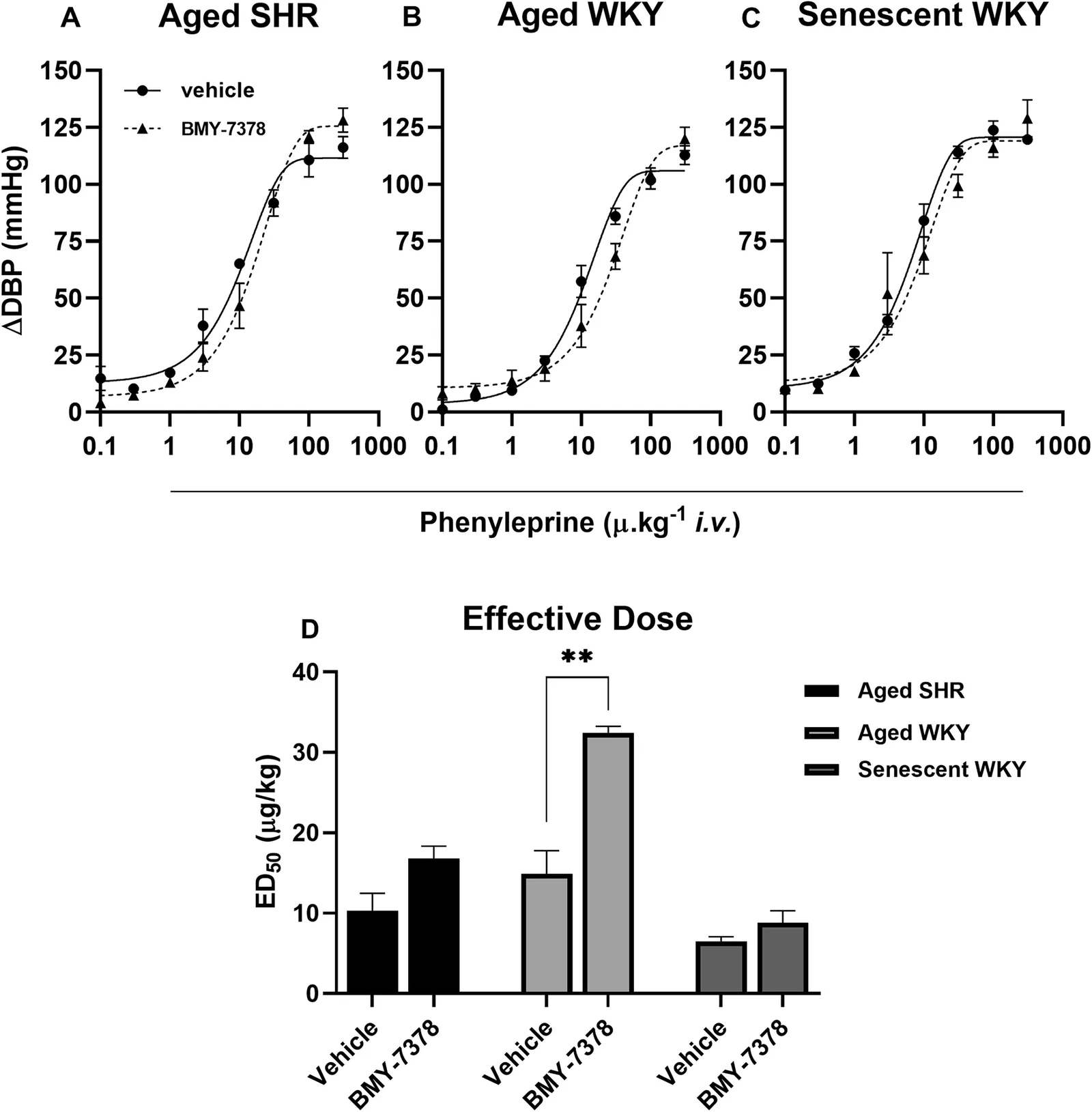
α_1D_-adrenoceptor blockade with BMY-7378 reduces phenylephrine sensitivity in WKY rats. Phenylephrine dose-response curves in pithed **A** SHR and **B** WKY rats before and after BMY-7378 (1 mg/kg, i.v.). **C** Senescent WKY rats before and after BMY-7378 (1 mg/kg, i.v.). **D** ED_50_ values showing a significant increase (reduced sensitivity) after BMY-7378. Data are mean ± SEM. **p < 0.01 *vs*. respective control

In aged WKY rats, administration of the α_1D_-adrenoceptor antagonist BMY-7378 induced a significant rightward shift in the PE dose-response curve, as evidenced by an increase in ED_50_ from 10.9 ± 5.0 to 32.5 ± 1.4 µg/kg (*p* < 0.05). In contrast, BMY-7378 did not significantly modify the PE response in aged SHR rats (ED_50_: 16.8 ± 2.6 *vs*. 10.3 ± 3.8 µg/kg, *p* > 0.05; Fig. 3).

### Isolated aortic ring

#### Effect of corticosterone on isolated aortic rings

Pre-incubation with corticosterone significantly reduced PE sensitivity (pD_2_) in SHR rats, as evidenced by a rightward shift of the concentration-response curve (Supplementary Fig. 3). Interestingly, in aged WKY rats, corticosterone did not modify PE sensitivity, but significantly increased the maximal contractile response (E_max_) to PE, but it did not modify any of these parameters in young or senescent WKY rats (Supplementary Fig. 4).

**Fig. 4:**
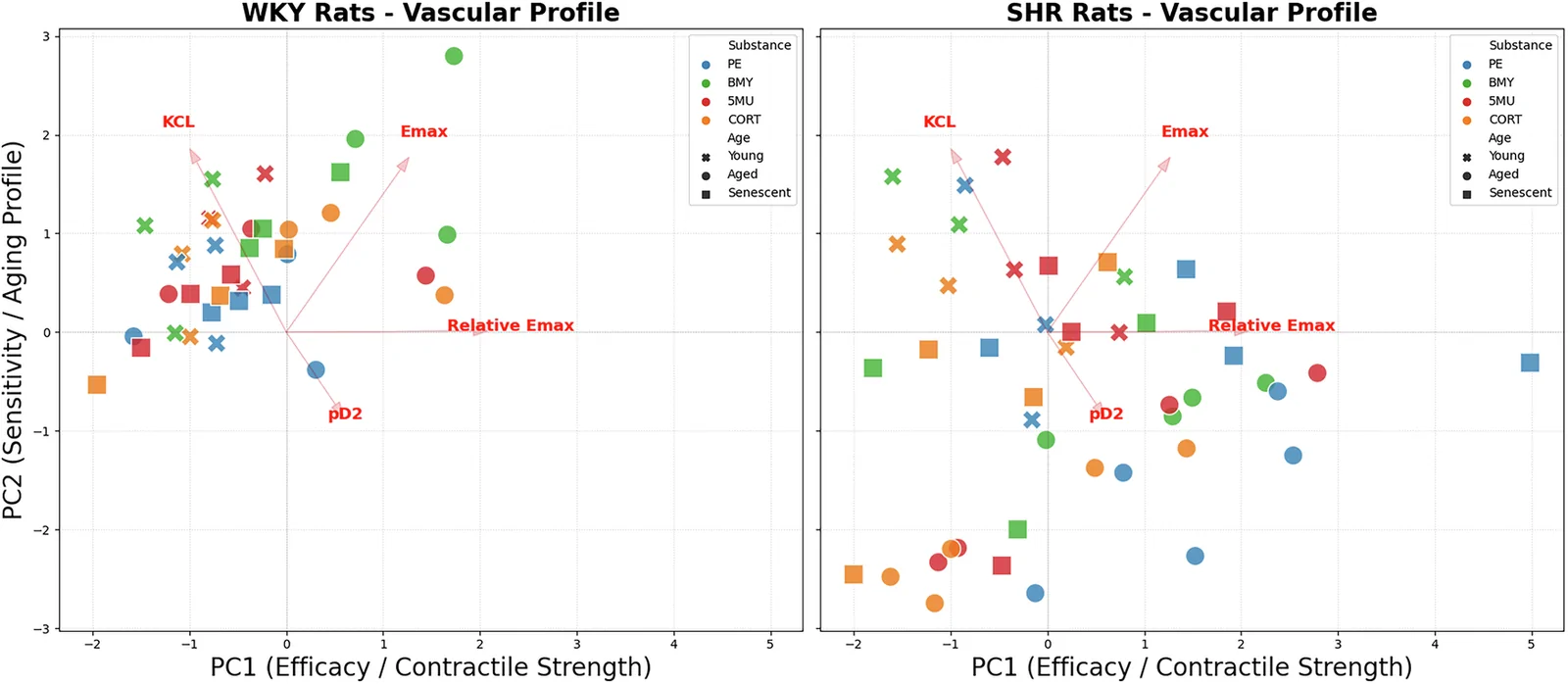
Principal Component Analysis (PCA) of vascular reactivity in WKY and SHR aortas. Multivariate integration of pharmacodynamic parameters. 2D-biplots representing the vascular functional profile of WKY (left) and SHR (right) rats across different life stages: Young (X), Aged (•), and Senescent (◼). The spatial distribution of data points is defined by the first two principal components, which capture the highest variance in efficacy/contractile strength (PC1) and sensitivity/aging profile (PC2). Red vectors indicate the loadings of the primary variables: pD_2_ (potency), E_max_ (maximal efficacy), KCl (structural contractile capacity), and Relative Emax (efficacy normalized to KCl). Colors represent the pharmacological modulators used: PE (blue), Corticosterone (CORT, orange), and the selective α_1D_-adrenoceptor antagonists BMY-7378 (BMY, green) and 5-Methylurapidil (5MU, red). Total Explained Variance: 76.30%

PCA revealed distinct vascular contraction profiles depending on strain, age, and pharmacological intervention. Young rats of both strains exhibited a contraction profile primarily dependent on KCl-induced depolarization. In contrast, aged and senescent SHR rats displayed a vascular profile associated with enhanced sensitivity to PE, as indicated by the location of blue points (PE) in the lower right quadrant of the plot, aligned with the pD_2_ vector (Fig. 4)

When comparing both strains, the normotensive WKY rats showed a more compact response pattern, reflected by the close clustering of data points. In contrast, SHR rats exhibited a more dispersed response, indicating greater heterogeneity in vascular reactivity within the hypertensive strain.

In the hypertensive model, corticosterone exerted a markedly more disruptive effect on the aging/sensitivity profile (PC2) compared to normotensive rats. This finding suggests that hypertension sensitizes the vasculature to the non-genomic effects of glucocorticoids.

Notably, the Euclidean distance between control (PE) and 5-methylurapidil (5-MU, α_1A_-AR selective antagonist) treatment was significantly larger in SHR than in WKY rats. This finding suggests a greater functional relevance of α_1A_-adrenoceptors in the hypertensive model.

Interestingly, the inhibition profile of corticosterone (CORT) followed a trajectory parallel to that of 5-MU in SHR, suggesting a convergent interference mechanism on the α_1A_-adrenoceptor pathway, which appears to be potentiated by the vascular pathology associated with hypertension (Fig. 4). All the concentration-response curves are in supplementary data 1 Figs. 5–7 and Tables 3–5.

Endothelial function was assessed by ACh-induced relaxation (10⁻⁹ M to 10⁻⁵ M). In SHR rats, relaxation was significantly reduced in senescent animals compared to young and aged groups. In contrast, WKY rats exhibited increased relaxation in senescent animals, as reflected by a greater area under the curve (Supplementary Figs. 8 and 9).

#### Pharmacodynamic analysis of antagonist co-incubations

To quantitatively assess the interaction between corticosterone and selective adrenergic antagonists, we calculated the percentage change in the area under the curve (%ΔAUC) using the formula:

$${Inh}={AUC}\,{Pe}-{AUC}\,{inhibitor}$$

$$\Delta {{\rm{AUC}}}( \% )=\frac{\left({In}h\,{Combination}-\left(\,{In}h\,{cort}+\,{In}h\,{bmy}\,{or}\,5{mu}\right)\right)}{{AUC}\,{PE}}\,{x}\,100$$

In contrast with the PCA analysis the %ΔAUC revealed distinct interaction patterns. In aged WKY rats (WKY-Aged), the co-incubation of CORT with BMY-7378 resulted in a greater additive inhibition of the phenylephrine response ( + 51.9%). Conversely, in senescent SHR (SHR-SEN), the heatmap showed a negative %ΔAUC for both α_1A_- and α_1D_-AR pathways (approximately −50%). Furthermore, the deviation from additivity in the α_1A_-AR pathway was minimal in WKY rats, but markedly antagonistic in SHR aged and senescent, indicating that young SHR did not have this adrenergic remodeling (Supplementary Data 1, Fig. 10).

### Adrenal index

The adrenal index, calculated as: *Adrenal index* = (adrenal weight (mg)/body weight (g)) × 100, provides a relative measure of adrenal size in relation to body mass, offering insights into adrenal activity and potential modulation of corticosterone production.

Strain-dependent differences in serum corticosterone concentrations were detected exclusively in young animals, where SHR showed significantly higher values than WKY rats (Supplementary Data 1, Table 6).

### Correlation analysis

**Fig. 5:**
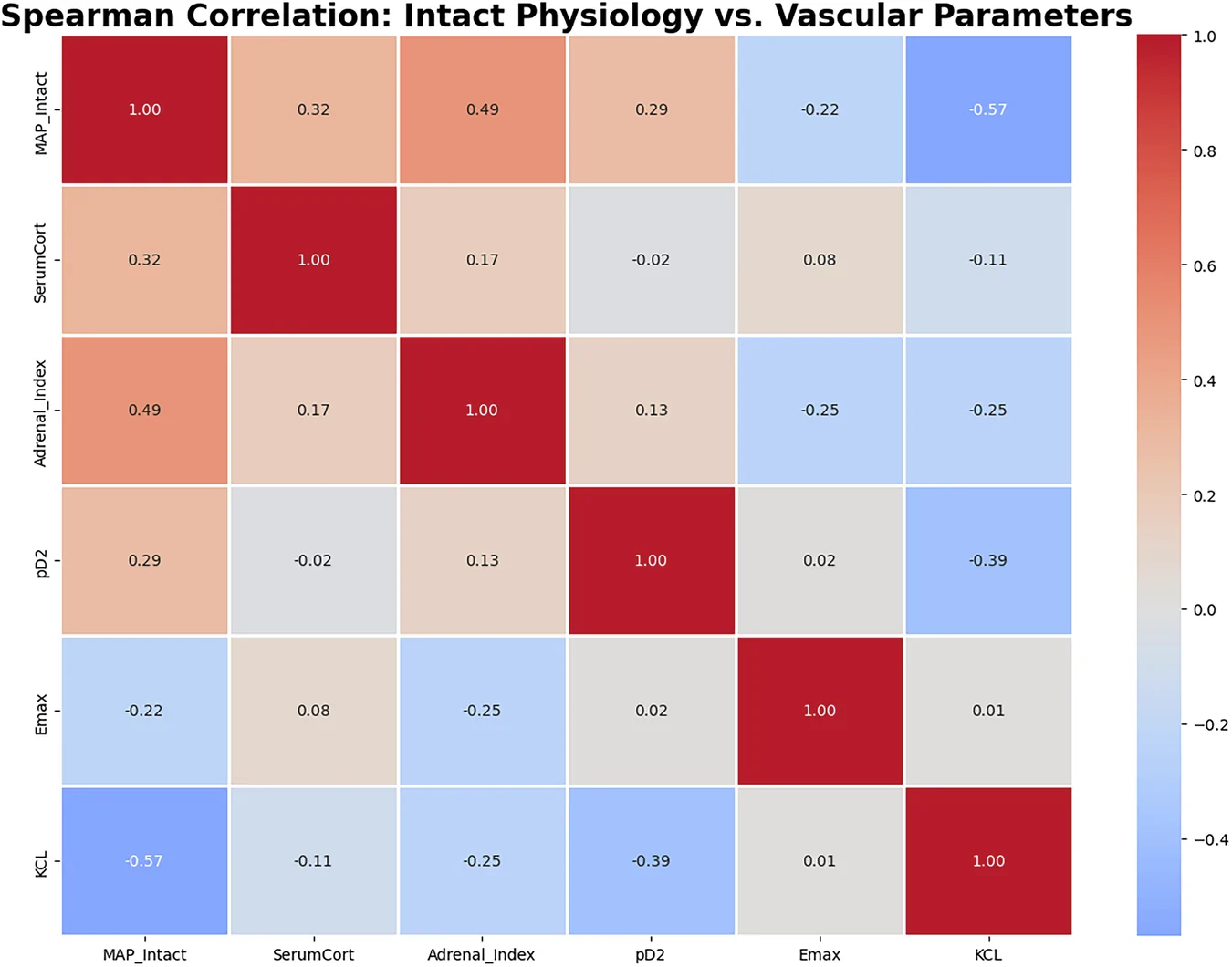
Spearman Correlation: Intact Physiology vs. Vascular Parameters. Heat map showing pairwise Spearman correlation coefficients (r) for MAP_Intact, serum corticosterone, adrenal index, pD_2_, Emax, and KCl-induced contraction. Data were pooled from all experimental groups. Red indicates positive correlations, blue negative correlations, with color intensity proportional to r. MAP mean arterial pressure, pD_2_ −log EC_50_

Spearman correlation analysis revealed that systemic hypertension in the not-pithed state (MAP_Intact), was strongly associated with chronic stress markers and vascular remodeling. Specifically, a robust positive correlation was found between MAP_Intact and Adrenal_Index (*r* = 0.49, *p* < 0.05). Interestingly, a strong negative correlation between MAP_Intact and KCl-induced contraction (*r* = -0.57, *p* < 0.01) suggests structural vascular impairment associated with chronic pressure overload (Fig. 5).

### Molecular docking

The α_1A_- and α_1D_-adrenoceptors were selected as molecular docking targets based on their strong functional association identified by STRING-db analysis (version 12.5; [35]). The protein-protein interaction network revealed shared interactions with multiple Gq/11 protein subunits, highlighting their overlapping signaling pathways and co-expression in vascular smooth muscle cells.

The docking protocol was validated by redocking the co-crystallized ligand into the α_1A_-adrenoceptor structure. The resulting RMSD was within the acceptable range ( < 2.0 Å), confirming the reliability of the methodology [36].

**Fig. 6:**
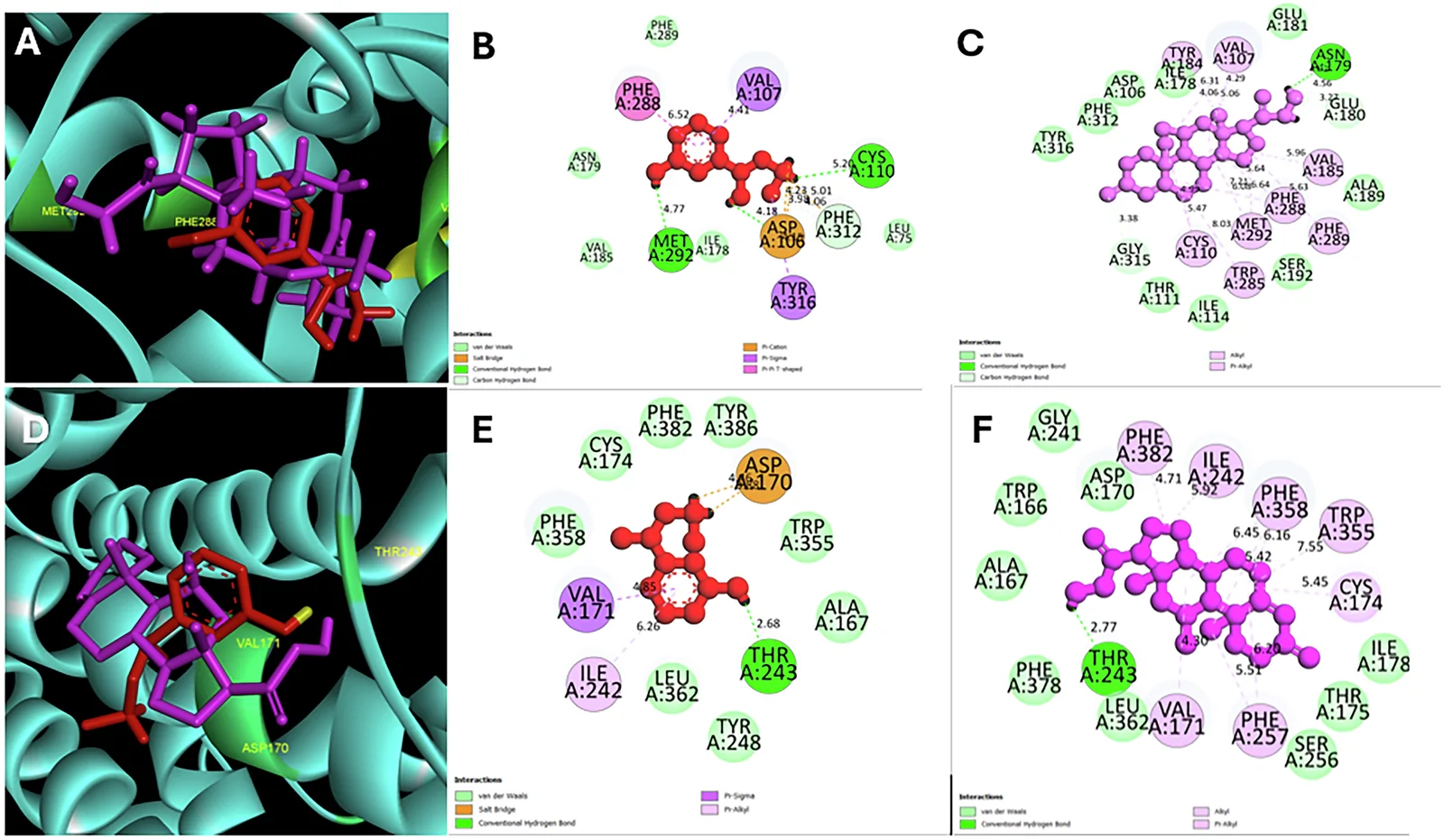
Docking Visualization. **A** Tree-dimensional view of amino acid residues involved in α_1A_-adrenoceptor activation. **B** Two-dimensional interaction diagram of PE. **C** Two-dimensional interaction diagram of corticosterone. Ligands are presented in red (PE) and pink (corticosterone). **D** Tree-dimensional view of amino acid residues involved in α_1D_-adrenoceptor activation. **E** Two-dimensional interaction diagram of PE. **F** Two-dimensional interaction diagram of corticosterone. Ligands are represented in red (PE) and pink (corticosterone)

PE was initially docked into both receptor subtypes to establish a reference binding profile (Supplementary Data 3, Table 1). For the α_1A_-adrenoceptor, PE engaged in characteristic polar and π–cation interactions with key residues (Asp106, Met292, Phe312), consistent with the conserved orthosteric binding mode of aminergic ligands [26]. In contrast, corticosterone docked into the same cavity, forming extensive π–alkyl interactions (Met292, Tyr184, Phe288) and occupying the orthosteric site. Corticosterone exhibited a higher binding affinity for the α_1A_-adrenoceptor than PE (ΔG = -9.14 *vs*. -6.49 kcal/mol; Ki = 198.13 nM *vs*. 17.36 µM), supporting a competitive displacement mechanism that may explain the rightward shift of PE dose-response curves observed in SHR rats (Fig. 6).

Docking into the α_1D_-adrenoceptor showed that PE interacted with Asp170 and Val171, as previously reported [37,38] Corticosterone also bound to the α_1D_-orthosteric pocket with multiple contacts (Thr243, Ile242, Phe382, Phe358), showing a similar binding affinity to PE (ΔG = -7.49 *vs*. -6.28 kcal/mol; Ki = 3.25 µM *vs*. 24.88 µM). However, no functional rightward shift was observed in WKY rats, where the α_1D_-receptor predominates, suggesting that corticosterone occupies the α_1D_-site without effective antagonism.

## Discussion

An important observation from this study is the discrepancy between the lack of effect of acute corticosterone on blood pressure in conscious rats, and the significant hypotensive effects observed in pithed preparations. Several factors may explain this difference. First, in conscious rats, compensatory baroreflex mechanisms would be expected to counteract any direct vasodilatory effect of corticosterone. In the pithed rat preparation, the central nervous system is destroyed by pithing, which eliminates supraspinal and baroreflex influences [21]. This allows the direct vascular effects of drugs to be observed without compensatory autonomic responses. Second, in conscious animals, endogenous sympathetic tone and circulating catecholamines may mask or counteract the vasodilatory effects of corticosterone. Third, the HPA axis and sympathetic nervous system exhibit complex bidirectional interactions; glucocorticoids can modulate central sympathetic outflow, which may obscure peripheral vascular effects. Fourth, the tail-cuff method measures blood pressure in the caudal artery, which may have different adrenergic receptor profiles compared to the carotid or other vascular beds. Thus, the hypotensive effects observed in pithed rats likely reflect a direct vascular action of corticosterone that is buffered by intact physiological regulatory mechanisms in conscious animals. This observation underscores the importance of using multiple experimental preparations to fully characterize the vascular effects of glucocorticoids.

The observation that acute corticosterone administration decreased phenylephrine sensitivity (increased ED_50_) (Fig. 1) in pithed rats, and reduced contractile responses in isolated aortic rings from SHR (Fig. 2), points to a direct, nongenomic vasodilator effect of this glucocorticoid in the hypertensive vasculature. This interpretation is strongly supported by the work of English et al.[39], who demonstrated that cortisol, the human equivalent of rodent corticosterone, produces an acute, dose-dependent vasodilatation in isolated coronary and pulmonary arteries from Wistar rats. Notably, this vasodilatory response occurred within seconds to minutes, consistent with a nongenomic mechanism of action. In their study, cortisol was the least potent vasodilator among the steroids tested, yet it consistently relaxed pre-contracted vessels. Our findings extend this concept by showing that in the SHR strain, characterized by genetic hypertension and vascular remodeling, corticosterone not only induces vasodilation, but also specifically desensitizes α_1_-adrenoceptor-mediated vasoconstriction, an effect that was absent in normotensive WKY rats. Thus, the hypertensive phenotype may sensitize the vasculature to the vasodilatory actions of glucocorticoids, a possibility that warrants further investigation into the molecular crosstalk between glucocorticoid and adrenergic signaling pathways.

The loss of the corticosterone-induced hypotensive effect and the absence of phenylephrine desensitization in senescent SHR (12 months), while preserved in young (3 months) and aged (6 months) animals, is likely explained by an age-related decline in the vascular α_1A_-adrenoceptor. Arce and colleagues [13] demonstrated that the α_1A_/nNOS-mediated desensitization mechanism, which acts as a physiological brake against excessive vasoconstriction, is absent in aged animals, coinciding with a significant reduction in α_1A_-adrenoceptor expression in the aorta. Therefore, the absence of a functional response to corticosterone, in our senescent SHR model, is consistent with the loss of its primary vascular target, the α_1A_-adrenoceptor. Importantly, the effect is preserved at 6 months (aged) but lost at 12 months (senescent), suggesting a progressive decline in this pathway with advancing age. This age-dependent decline was not only observed in rats, but was further corroborated by a negative correlation between age and α_1A_-adrenoceptor mRNA concentrations. in the left ventricle of human heart failure patients, suggesting a clinically relevant phenomenon. It is important to note that while we assessed endothelial function through acetylcholine (ACh)-induced relaxation in our aortic ring preparations (Supplementary Figs. 8 and 9), we did not directly measure nNOS activity, NO availability, or perform endothelial denudation experiments. The α_1A_-AR/nNOS pathway is therefore an inferred mechanism based on prior literature [13], rather than a directly tested pathway in this study. However, our ACh relaxation data provide relevant context: in SHR rats, senescent animals exhibited significantly reduced relaxation compared to young and aged groups, suggesting progressive endothelial dysfunction with advancing age in the hypertensive strain. In contrast, WKY rats showed the opposite pattern, with senescent animals displaying the greatest area under the curve in ACh relaxation assays. These strain-specific differences in endothelial function may contribute to the differential vascular responses to corticosterone, observed between SHR and WKY rats, and between age groups. Future studies employing NOS inhibition (e.g., L-NAME), endothelial denudation, or nNOS-specific pharmacological or genetic approaches would be necessary to definitively establish the contribution of the nNOS pathway to corticosterone-induced desensitization.

Serum corticosterone was measured only under basal conditions, and we acknowledge that post-administration concentrations were not assessed in this study. This represents a limitation, as we cannot directly correlate the achieved plasma concentrations with the observed functional effects. Nevertheless, the dose of corticosterone used in our experiments (5 mg/kg, i.p.) was selected based on previous studies, demonstrating that this dose produces rapid physiological effects in rodents, and mimics plasma concentrations observed during substantial stress [18].

Based on the functional vascular data obtained in pithed rats, we next characterized the α_1_-adrenoceptors present in the aortic vasculature, using isolated organ bath experiments. In these assays, SHR rats exhibited a predominant functional profile for the α_1A_-adrenoceptor, based on the Euclidean distance between PE and 5-MU treatment was significantly larger in SHR than in WKY rats (Fig. 4). This finding suggests a greater functional relevance of α_1A_-adrenoceptors in the hypertensive model.

Furthermore, hypertension was associated with an impaired contractile response to the depolarizing stimulus KCl (Fig. 5), suggesting that chronic pressure overload leads to structural or functional vascular remodeling, that reduces the intrinsic contractile capacity of the smooth muscle. In agreement with this observation, Clark and colleagues [40] reported that maximal isometric tension development in response to 50 mmol/L KCl, was significantly lower in aortic rings from SHR compared to WKY rats (49.2 ± 2.9 vs. 64.4 ± 1.2 mN/mm²), despite the presence of significant vascular hypertrophy in the hypertensive group. These authors proposed that this reduced force-generating capacity may reflect a fundamental alteration in the contractile machinery of SHR vascular smooth muscle, possibly related to changes in calcium handling or myosin light chain phosphorylation, rather than simply being a consequence of increased wall mass.

Given the increased functional contribution of α_1A_-adrenoceptors in SHR, we performed PCA to explore the impact of corticosterone on the vascular profile of this strain. The PCA revealed that in SHR, corticosterone-treated rings clustered together with 5-MU, suggesting that the glucocorticoid acts through pathways aligned with the predominant α_1A_-AR mediated mechanism, rather than introducing a distinct vasoactive signature. This observation raised the hypothesis that corticosterone may directly interact with the α_1A_-adrenoceptor or its downstream signaling pathways. While our data strongly suggest that corticosterone interacts with the α_1A_-adrenoceptor, several alternative mechanisms could contribute to the observed effects and should be considered. First, the functional overlap between corticosterone and 5-methylurapidil observed in PCA does not definitively prove direct receptor antagonism; it could also reflect convergence at downstream signaling pathways, such as Gq/_11_-mediated phospholipase C activation or calcium mobilization. Second, corticosterone could modulate endothelial NO pathways, either through nNOS (as previously described for α_1A_-ARmediated desensitization) or through endothelial NOS, which is known to be influenced by glucocorticoids. Third, non-genomic membrane effects of steroids, including changes in membrane fluidity or interactions with membrane-bound receptors such as GPER1 (G protein-coupled estrogen receptor), could contribute to the observed vasodilation. Fourth, glucocorticoids may affect calcium handling in vascular smooth muscle cells through rapid modulation of ion channels or intracellular calcium stores. While our molecular docking data support a direct interaction between corticosterone and the α_1A_-adrenoceptor orthosteric site, definitive proof of direct antagonism in intact vascular tissue would require additional experiments, such as radioligand binding assays or functional studies with specific α_1A_-AR mutants. We therefore conclude that corticosterone-induced desensitization is likely mediated through α_1A_-adrenoceptor-dependent mechanisms, but alternative pathways cannot be excluded.

Notably, a significant body of evidence supports a close and bidirectional interaction between corticosterone and α_1_-adrenoceptors in the central nervous system. In 1985, Duman and colleagues [15] demonstrated that corticosterone induces desensitization of central α_1_-adrenergic receptors, a phenomenon that correlates with depressive-like behaviors in animal models, suggesting that glucocorticoids can directly modulate the functional state of these receptors. Subsequent research has reinforced this view, proposing that α_1_-adrenoceptor desensitization may represent a protective mechanism against stress, acting as a preventive brake against excessive sympathetic nervous system-mediated damage [41]. Similarly, Campeau et al. [16] demonstrated that stress exposure rapidly upregulates α_1D_-adrenoceptor mRNA expression in the dentate gyrus, reinforcing the idea that glucocorticoids and noradrenergic systems are functionally intertwined at the molecular level. While these studies were conducted in the central nervous system, they provide a robust conceptual framework for our peripheral findings, establishing that α_1_-adrenoceptors are not static entities but are dynamically regulated by glucocorticoid concentrations. Our correlation analysis revealed that in intact (non-pithed) rats, MAP was positively correlated with adrenal index (r = 0.49, *p* < 0.05), indicating that heightened hypothalamic-pituitary-adrenal axis activity is associated with elevated BP in the hypertensive state (Fig. 5). Extending this paradigm to the vascular wall, our results suggest that in the SHR aorta, the α_1A_-adrenoceptor is particularly sensitive to modulation by corticosterone, likely due to the vascular remodeling and altered adrenergic receptor hierarchy characteristic of the hypertensive state.

To test this hypothesis, we performed co-incubation experiments with the selective antagonists BMY-7378 (α_1D_) and 5-MU (α_1A_), and calculated the percentage deviation from additivity (Δ%). This analysis revealed that in SHR, corticosterone reduced the antagonist effect of 5-MU by ~50%, as evidenced by a negative ΔAUC% value, indicating a competitive or functional antagonism between corticosterone and the α_1A_-AR blocker. In contrast, this effect was markedly attenuated in WKY rats, suggesting that the α_1A_-adrenoceptor is not the predominant signaling pathway in the normotensive strain. This finding aligns with the observation that in pithed rats, the α_1D_-AR selective antagonist BMY-7378 produced a significant rightward shift of the phenylephrine dose-response curve exclusively in aged WKY rats, with no such effect observed in SHRs. In agreement with this, Villalobos-Molina and colleagues demonstrated that in aged WKY rats BMY-7378 induces a substantial displacement of the phenylephrine pressor response (dose ratio ~3.2), confirming the functional relevance of α_1D_-adrenoceptors in the normotensive vasculature at this age. Conversely, the same study reported that young WKY rats failed to exhibit this shift, further supporting that the α_1D_-mediated component emerges with maturation [10].

To directly test whether corticosterone can physically interact with the α_1A_-adrenoceptor, we performed molecular docking simulations. The results demonstrated that corticosterone binds to the orthosteric binding site of the α_1A_-adrenoceptor, with favorable binding energy. This computational data supports the functional evidence that corticosterone acts as a direct modulator of α_1A_-adrenoceptor function, competing with endogenous agonists and pharmacological antagonists for the same receptor binding pocket.

These findings support a model where strain-specific α_1_-adrenoceptor expression determines glucocorticoid sensitivity. In SHR, greater functional relevance of α_1A_-adrenoceptors (evidenced by PCA, 5-MU sensitivity, and docking) enhances corticosterone-mediated desensitization. This effect is age-dependent, maximal in aged SHR and lost in senescent animals, consistent with the age-related decline of the α_1A_-AR/nNOS pathway [13]. In contrast, normotensive WKY rats rely predominantly on α_1D_-adrenoceptors, which are insensitive to corticosterone. Collectively, we identify corticosterone as an endogenous modulator of vascular tone acting through α_1A_-adrenoceptors, revealing a novel glucocorticoid–adrenergic interaction that may contribute to stress-related BP variability, and the paradoxical hypotension observed in some hypertensive patients, particularly with aging.

In conclusion, our findings demonstrate that corticosterone selectively desensitizes α_1_-adrenoceptor-mediated vasoconstriction in SHR rats, an effect that is preserved in young and aged but lost in senescent animals. Molecular docking and pharmacological evidence strongly suggest that this effect involves high-affinity binding to the α_1A_-adrenoceptor, although alternative mechanisms may also contribute. This strain-specific, age-dependent interaction reveals a novel non-genomic glucocorticoid action on the vasculature, which may have implications for understanding blood pressure regulation in hypertension and during aging.

Supplementary information is available at Hypertension Research’s website.

## Supplementary information

Supplementary data 1

Supplementary data 1

Supplementary data3
