# Supplementary material for: Corticosterone binds α1A-adrenoceptors with high affinity and induces strain-dependent vascular desensitization in female spontaneously hypertensive rats
Source: Hypertens Res. 2026 Aug 6;49(10):2803–16. doi: 10.1038/s41440-026-02760-z (PMC13634920; doi:10.1038/s41440-026-02760-z)
Supplement: Supplementary file 1 [file 41440_2026_2760_MOESM1_ESM.docx]

**Corticosterone binds α1A-adrenoceptors with high affinity and induces strain-dependent vascular desensitization in female spontaneously hypertensive rats**

Yanina A. Santander-Plantamura^a**^, Karen Nuñez-Solano^b^, Santiago C. Sigrist-Flores^a,c^, Itzell A. Gallardo-Ortíz^a^, Enrique Hong^d^, Rafael Villalobos-Molina^a*^

a. Unidad de Investigación en Biomedicina, Facultad de Estudios Superiores Iztacala, Universidad Nacional Autónoma de México, Tlalnepantla, Edo. de México, México.
b. Carrera de Químico Farmacéutico Biólogo, Facultad de Estudios Superiores Zaragoza, Universidad Nacional Autónoma de México, Iztapalapa, Ciudad de México, México.
c. Carrera de Médico Cirujano, Facultad de Estudios Superiores Iztacala, Universidad Nacional Autónoma de México, Tlalnepantla, Edo. de México, México.
d. Departamento de Farmacobiología, Centro de Investigación y de Estudios Avanzados, Sede Sur, Ciudad de México, México.

* correspondence author: Rafael Villalobos-Molina

** co-correspondence author: Yanina A. Santander-Plantamura

Unidad de Investigación en Biomedicina, Facultad de Estudios Superiores Iztacala Universidad Nacional Autónoma de México,

Ave. de Los Barrios 1, Los Reyes Ixtacala, Tlalnepantla, 54090. Estado de México, México

Tel. +52 5623-1333 ext 39795

Email: [villalobos@unam.mx](mailto:villalobos@unam.mx); [santander.plantamura@gmail.com](mailto:santander.plantamura@gmail.com);

**Supplementary data 1**

**Supplementary Table 1.** **Systolic (SBP), Mean (MAP), and Diastolic (DBP) Blood Pressure in All Experimental Groups (mmHg)**

|  | SBP | MAP | DBP |
| --- | --- | --- | --- |
| WKY Young | 113 ± 10 | 95 ± 10 | 86 ± 10 |
| SHR Young | 147 ± 10 ^a^ | 125 ± 7 ^a^ | 116 ± 9 ^a^ |
| WKY Aged | 123 ± 10 | 94 ± 10 | 91 ± 11 |
| SHR Aged | 148 ± 6 ^b^ | 121 ± 13 ^b^ | 116 ± 10 ^b^ |
| WKY Senescent | 114 ± 7 | 87 ± 5 | 75 ± 8 |
| SHR Senescent | 150 ± 5 ^c^ | 128 ± 10 ^c^ | 117 ± 10 ^c^ |

^a^p < 0.05 *vs.* WKY Young, ^b^p < 0.05 *vs.* WKY Aged, ^c^p < 0.05 *vs.* WKY Senescent.

**Supplementary Table 2.** Cumulative dose-response curves to phenylephrine (0.1 – 310 µg/kg, i.v.). ED_50_ (the dose producing 50% of the maximal pressor response), and E_max_ (the maximal increase in diastolic blood pressure, ΔmmHg) were calculated by non-linear regression analysis for each individual curve. Data are presented as mean ± SEM for each experimental group. In a separate set of experiments, rats received an intraperitoneal injection of corticosterone (5 mg/kg) 30 minutes prior to the phenylephrine curve, to evaluate acute glucocorticoid modulation of vascular reactivity. Parameters of phenylephrine dose-response curves in pithed female rats under control conditions and after acute corticosterone administration.

|  | ED_50_ (µg/kg) | E_max_ (mmHg) | ED_50_  (µg/kg) | E_max_  (mmHg) | ED_50_  (µg/kg) | | E_max_  (mmHg) |
| --- | --- | --- | --- | --- | --- | --- | --- |
| Group | **Young** | | **Aged** | | **Senescent** | | |
| WKY | 29.2 ± 1.0 | 120 ± 3 | 10.9 ± 5.0* | 116 ± 14 | | 7.5 ± 2.0*^,%^ | 117 ± 5 |
| SHR | 4.6 ± 1.2^a^ | 127 ± 6 | 10.3 ± 3.8^&^ | 122 ± 21 | | 6.5 ± 1.0 | 124 ± 1 |
| Corticosterone 5 mg/kg i.p. | | | | | | | |
| WKY | 19.3 ± 5.7 | 126 ± 11 | 12.7 ± 5.6 | 117 ± 5 | | 8.6 ± 0.3 | 135 ± 1 |
| SHR | 10.9 ± 1.3 ^#^ | 120 ± 4 | 20.0 ± 0.2 ^#^ | 127 ± 4 | | 7.9 ± 2.1 | 142 ±19 |

Data are expressed as mean ± SEM.

^a^p < 0.05 *vs*. WKY, ^#^p < 0.05 *vs*. SHR without corticosterone.

^&^ p < 0.05 *vs.* SHR young, *p < 0.05 *vs*. WKY young, ^%^ p < 0.01 *vs.* WKY Aged

**Supplementary Figure 1. Complete phenylephrine dose-response curves in pithed female rats under control conditions.**

Full cumulative dose-response curves to phenylephrine (0.1 – 310 µg/kg, i.v.) in pithed female rats. Left panels show curves under control conditions: **(A)** SHR rats at young, aged, and senescent ages; **(B)** WKY rats at young, aged, and senescent ages. Data are expressed as mean ± SEM (n = 3-5 per group). Curves were fitted by non-linear regression. Corresponding ED_50_ and Emax values are presented in Supplementary Table 2.

**Supplementary Figure 2. Phenylephrine-induced contractile responses in isolated aortic rings from young, aged, and senescent rats.**

Concentration-response curves to phenylephrine (10^-9^ M – 10^-4^ M) were evaluated in isolated aortic rings from **(A)** SHR and **(B)** WKY rats at three different ages: young (3 months), aged (6 months), and senescent (12 months). Data are presented as mean ± SEM (n = 3-5 per group). Corresponding pD₂ and Eₘₐₓ values are presented in Supplementary Tables 2, 3 and 4.

**Supplementary Figure 3. Effect of corticosterone on phenylephrine-induced contractile responses in isolated aortic rings from SHR rats.**

Concentration-response curves to phenylephrine (10^-9^ M – 10^-4^ M) were evaluated in isolated aortic rings from female rats in the absence (vehicle) and presence of corticosterone (CORT, 10^-6^ M). **(A)** SHR Young, **(B)** SHR Aged, **(C)** SHR Senescent. Data are presented as mean ± SEM (n = 3-5 per group). Curves were fitted by non-linear regression.

**Supplementary Figure 4. Effect of corticosterone on phenylephrine-induced contractile responses in isolated aortic rings from WKY rats.**

Concentration-response curves to phenylephrine (10^-9^ M – 10^-4^ M) were evaluated in isolated aortic rings from female rats in the absence (vehicle) and presence of corticosterone (CORT, 10^-6^ M). Experiments were performed in **(A)** WKY young **(B)** WKY Aged, **(C)** WKY senescent. Contractions are expressed as g. Data are presented as mean ± SEM (n = 3-5 per group). Curves were fitted by non-linear regression.

**Supplementary Figure 5. Effect of selective α₁_A_- and α₁_D_-adrenoceptor blockade on phenylephrine-induced contraction in isolated aortic rings from aged rats.**

Concentration-response curves to phenylephrine (10^-9^ M – 10^-4^ M) in isolated aortic rings from aged (6 months) female rats under control conditions and after pre-incubation with the selective α₁_A_-adrenoceptor antagonist 5-methylurapidil (10^-7^ M) or the selective α₁_D_-adrenoceptor antagonist BMY-7378 (10^-7^ M). Experiments were performed in (A) SHR Young, (B) WKY Young. Contractions are expressed as g. Data are presented as mean ± SEM (n = 3-5 per group). Curves were fitted by non-linear regression.

**Supplementary Figure 6. Effect of selective α₁_A_- and α₁_D_-adrenoceptor blockade on phenylephrine-induced contraction in isolated aortic rings from aged rats.**

Concentration-response curves to phenylephrine (10^-9^ M – 10^-4^ M) in isolated aortic rings from aged (6 months) rats under control conditions and after pre-incubation with the selective α₁_A_-adrenoceptor antagonist 5-methylurapidil (10^-7^ M), or the selective α₁_D_-adrenoceptor antagonist BMY-7378 (10^-7^ M). Experiments were performed in (A) SHR Aged, (B) WKY Aged. Contractions are expressed as g. Data are presented as mean ± SEM (n = 3-5 per group). Curves were fitted by non-linear regression.

**Supplementary Figure 7. Effect of selective α₁A- and α₁_D_-adrenoceptor blockade on phenylephrine-induced contraction in isolated aortic rings from Senescent rats.**

Concentration-response curves to phenylephrine (10^-9^ M – 10^-4^ M) in isolated aortic rings from senescent (12 months) rats under control conditions and after pre-incubation with the selective α₁_A_-adrenoceptor antagonist 5-methylurapidil (10^-7^ M) or the selective α₁_D_-adrenoceptor antagonist BMY-7378 (10^-7^ M). Experiments were performed in (A)  WKY Senescent, (B)  SHR Senescent. Contractions are expressed as g. Data are presented as mean ± SEM (n = 3-5 per group). Curves were fitted by non-linear regression.

**Supplementary Table 3. Pharmacodynamic parameters of phenylephrine-induced contraction in isolated aortic rings from aged rats.**

Concentration-response curves to phenylephrine (10^-9^M – 10^-4^ M) were evaluated in isolated aortic rings from aged (6 months) WKY and SHR, and in the presence of various pharmacological modulators. pD₂ (−log EC₅₀) and Eₘₐₓ (maximal contraction ∆g) were calculated by non-linear regression analysis. Data are expressed as mean ± SEM (n = 3-5 per group). Abbreviations: PE, Phenylephrine, BMY, BMY-737; 5-MU, 5-methylurapidil; CORT, corticosterone.

| Experimental condition | SHR |  | WKY |  |
| --- | --- | --- | --- | --- |
|  | **E_max_ (**∆**g)** | **pD₂** | **E_max_ (**∆**g)** | **pD₂** |
| PE | 1.63 ± 0.40 | 7.21 ± 0.62 | 1.72 ± 0.33 | 6.44 ± 0.50 |
| + CORT | 1.18 ± 0.45 | 6.52 ± 0.32^#^ | 2.17 ± 0.07 ^#^ | 6.38 ± 0.34 |
|  |  | **(ΔpD₂: -0.71)** |  | **(ΔpD₂:-0.06 )** |
| + BMY | 1.71 ± 0.26 | 5.59 ± 0.67 ^#^ | 2.57 ± 0.32^#^ | 5.52 ± 0.32^##^ |
|  |  | **(ΔpD₂: -1.63)** |  | **(ΔpD₂: -0.92)** |
| + CORT + BMY | 1.25 ± 0.60 | 5.58 ± 0.15 ^#^ | 1.77 ± 0.34 | 5.39 ± 0.26^#^ |
|  |  | **(ΔpD₂:-1.63)** |  | **(ΔpD₂: -1.05 )** |
| + 5-MU | 1.43 ± 0.58 | 6.32 ± 0.21 ^#^ | 1.92 ± 0.33 | 5.96 ± 0.32^##^ |
|  |  | **(ΔpD₂: -0.85)** |  | **(ΔpD₂: -0.48)** |
| + CORT + 5-MU | 1.29 ± 0.52 | 6.57 ± 0.27 ^#^ | 2.12 ± 0.55 | 6.52 ± 0.38 |
|  |  | **(ΔpD₂:-0.64)** |  | **(ΔpD₂: 0.08)** |

^#^ p < 0.05, ^##^ p < 0.01 *vs.* PE.

**Supplementary Table 4. Pharmacodynamic parameters of phenylephrine-induced contraction in isolated aortic rings from young rats.**

Concentration-response curves to phenylephrine (10^-9^ M – 10^-4^ M) were evaluated in isolated aortic rings from young (3 months) WKY and SHR, and in the presence of various pharmacological modulators. pD₂ (−log EC₅₀) and Eₘₐₓ (maximal contraction expressed as ∆g) were calculated by non-linear regression analysis. Data are expressed as mean ± SEM (n = 3-5 per group). Abbreviations: PE, Phenylephrine, BMY, BMY-7378; 5-MU, 5-methylurapidil; CORT, corticosterone.

| Experimental condition | SHR |  | WKY |  |
| --- | --- | --- | --- | --- |
|  | **E_max_ (**∆**g)** | **pD₂** | **E_max_ (**∆**g)** | **pD₂** |
| PE | 1.81 ± 0.24 | 7.66 ± 0.82 | 1.74 ± 0.12 | 6.72 ± 0.27 |
| + CORT | 1.73 ± 0.05 | **6.48 ± 0.25^##^** | 1.74 ± 0.26 | 6.45 ± 0.19 |
|  |  | **(ΔpD₂:-1.13)** |  | **(ΔpD₂:- 0.27)** |
| + BMY | 1.89 ± 0.14 | **5.72** ± **0.32^#^** | 1.74 ± 0.25 | 5.92 ± 0.49^##^ |
|  |  | **(ΔpD₂:-1.48)** |  | **(ΔpD₂:-0.80 )** |
| + CORT + BMY | 2.00 ± 0.20 | 5.53 ± 0.05 ^##^ | 1.72 ± 0.16 | 5.83 ± 0.50^##^ |
|  |  | **(ΔpD₂:-1.16)** |  | **(ΔpD₂:-0.89 )** |
| + 5-MU | 2.01 ± 0.14 | 6.68 ± 0.49^##^ | 1.96 ± 0.19 | 6.27 ± 0.19 ^##^ |
|  |  | **(ΔpD₂: -0.77)** |  | **(ΔpD₂:-0.45 )** |
| + CORT + 5-MU | 1.90 ± 0.17 | 6.09 ± 0.26^#^ | 1.79 ± 0.11 | 6.25 ± 0.40^##^ |
|  |  | **(ΔpD₂:-0.70)** |  | **(ΔpD₂:- 0.47)** |

^#^ p < 0.05, ^##^ p < 0.01 *vs.* PE

**Supplementary Table 5. Pharmacodynamic parameters of phenylephrine-induced contraction in isolated aortic rings from senescent rats.**

Concentration-response curves to phenylephrine (10^-9^ M – 10^-4^ M) were evaluated in isolated aortic rings from senescent (12 months) WKY and SHR. pD₂ (−log EC₅₀) and Eₘₐₓ (maximal contraction expressed as ∆g) were calculated by non-linear regression analysis. Data are expressed as mean ± SEM (n = 3-5 per group). Abbreviations: PE, Phenylephrine; BMY, BMY-7378; 5-MU, 5-methylurapidil, CORT, corticosterone.

| Experimental condition | SHR |  | WKY |  |
| --- | --- | --- | --- | --- |
|  | **E_max_ (**∆**g)** | **pD₂** | **E_max_ (**∆**g)** | **pD₂** |
| PE | 2.16 ± 0.40 | 7.21 ± 0.40 | 1.76 ± 0.10 | 6.44 ± 0.26 |
| + CORT | 1.70 ± 0.39 | 6.12 ± 0.50 ^#^ | 1.44 ± 0.39 | 6.16 ± 0.20 |
|  |  | **(ΔpD₂: -0.85)** |  | **(ΔpD₂:-0.18)** |
| + BMY | 1.60 ± 0.49 | 6.12 ± 0.50^#^ | 2.06 ± 0.22 | 5.60 ± 0.25^##^ |
|  |  | **(ΔpD₂: -1.13)** |  | **(ΔpD₂: -0.84)** |
| + CORT + BMY | 1.81 ± 0.42 | 5.62 ± 0.27^#^ | 1.56 ± 0.02 | 5.25 ± 0.34^##^ |
|  |  | **(ΔpD₂: -1.59)** |  | **(ΔpD₂:-1.19)** |
| + 5-MU | 1.72 ± 0.61 | 6.34 ± 0.25^#^ | 1.60 ± 0.13 | 5.96 ± 0.13^##^ |
|  |  | **(ΔpD₂: -0.73)** |  | **(ΔpD₂:-0.48)** |
| + CORT + 5-MU | 2.11 ± 0.24 | 6.18 ± 0.34^#^ | 1.91 ± 0.25 | 5.74 ± 0.32^##^ |
|  |  | **(ΔpD₂:-1.03)** |  | **(ΔpD₂:-0.70)** |

^#^ p < 0.05 *vs.* PE, ^##^ p < 0.01 *vs.* PE

**Supplementary Figure 8**: Acetylcholine (ACh)-induced relaxation in isolated aortic rings from SHR and WKY rats. Concentration-response curves to ACh (10⁻⁹ M to 10⁻⁵ M) were evaluated in aortic rings pre-contracted with phenylephrine. Relaxation responses are expressed as percentage of pre-contraction. (A) SHR rats at young (3 months), aged (6 months), and senescent (12 months) ages. Data are presented as mean ± SEM (n = 3-5 per group). (B) Area under the curve (AUC). * *p < 0.05; **p < 0.05.*

**Supplementary Figure 9:** Acetylcholine (ACh)-induced relaxation in isolated aortic rings from SHR and WKY rats. Concentration-response curves to ACh (10⁻⁹ M to 10⁻⁵ M) were evaluated in aortic rings pre-contracted with phenylephrine. Relaxation responses are expressed as percentage of pre-contraction. (A) WKY rats at young, aged, and senescent ages. (B) AUC for all groups. Data are presented as mean ± SEM (n = 3-5 per group). * *p < 0.05; ** p < 0.01; *** p< 0.001.*

**Supplementary Figure 10. Heatmap of Pharmacological Interactions Between Corticosterone and Adrenergic Blockers.** Interaction Analysis of Vascular Reactivity to Phenylephrine (PE). The heatmap displays the deviation from additivity (∆%) in the Area Under the Curve (AUC), following pre-incubation with Corticosterone (CORT) and specific α1-adrenoceptor antagonists. Color Scale: Positive values (red) indicate synergistic potentiation of the inhibitory effect, where the combined action of CORT and the blocker exceeds the sum of their individual effects. Negative values (blue) represent competitive antagonism, indicating an interference between CORT and the blocker within the signaling pathway. Pathways: Columns represent the specific pathways for α_1D_ (blocked by BMY-7378) and α_1A_ (blocked by 5-Methylurapidil) receptor subtypes.

**Supplementary Table 6: Weight of adrenals glands and serum corticosterone.** Data are expressed as mean ± SEM. Abbreviation: WKY, Wistar Kyoto rats; SHR, spontaneously hypertensive rats.

| Group | Adrenal index | Corticosterone (ng/ml) |
| --- | --- | --- |
| WKY Young | 23 ± 4 | 778 ± 394 |
| SHR Young | 27 ± 6 | 1476 ± 224 * |
| WKY Aged | 18 ± 4 | 706 ± 324 |
| SHR Aged | 25 ± 6 | 524 ± 272^&&^ |

^*^p < 0.05 *vs*. WKY young, ^&&^ p < 0.01 *vs.* SHR young.
