# Supplementary material for: Corticosterone binds α1A-adrenoceptors with high affinity and induces strain-dependent vascular desensitization in female spontaneously hypertensive rats
Source: Hypertens Res. 2026 Aug 6;49(10):2803–16. doi: 10.1038/s41440-026-02760-z (PMC13634920; doi:10.1038/s41440-026-02760-z)
Supplement: Supplementary file 2 [file 41440_2026_2760_MOESM2_ESM.docx]

**Corticosterone binds α1A-adrenoceptors with high affinity and induces strain-dependent vascular desensitization in female spontaneously hypertensive rats**

Yanina A. Santander-Plantamura^a**^, Karen Nuñez-Solano^b^, Santiago C. Sigrist-Flores^a,c^, Itzell A. Gallardo-Ortíz^a^, Enrique Hong^d^, Rafael Villalobos-Molina^a*^

a. Unidad de Investigación en Biomedicina, Facultad de Estudios Superiores Iztacala, Universidad Nacional Autónoma de México, Tlalnepantla, Edo. de México, México.
b. Carrera de Químico Farmacéutico Biólogo, Facultad de Estudios Superiores Zaragoza, Universidad Nacional Autónoma de México, Iztapalapa, Ciudad de México, México.
c. Carrera de Médico Cirujano, Facultad de Estudios Superiores Iztacala, Universidad Nacional Autónoma de México, Tlalnepantla, Edo. de México, México.
d. Departamento de Farmacobiología, Centro de Investigación y de Estudios Avanzados, Sede Sur, Ciudad de México, México.

* correspondence author: Rafael Villalobos-Molina

** co-correspondence author: Yanina A. Santander-Plantamura

Unidad de Investigación en Biomedicina, Facultad de Estudios Superiores Iztacala Universidad Nacional Autónoma de México,

Ave. de Los Barrios 1, Los Reyes Ixtacala, Tlalnepantla, 54090. Estado de México, México

Tel. +52 5623-1333 ext 39795

Email: [villalobos@unam.mx](mailto:villalobos@unam.mx); [santander.plantamura@gmail.com](mailto:santander.plantamura@gmail.com);

**Supplementary Data 2 – Python script for PCA analysis of vascular reactivity profiles**

Python Script for PCA Analysis

Description: This script performs Z-score normalization and PCA to evaluate

aortic functional profiles across different age groups and inhibitors.

\# Import required libraries

import pandas as pd

from sklearn.decomposition import PCA

from sklearn.preprocessing import StandardScaler

\# 1. LOAD AND PREPROCESS

df = pd.read_excel('PCA.xlsx', sheet_name="sheet_1")

\# Clean column names

df.columns = [c.strip() for c in df.columns]

def process_groups_en(row):

    # Standardize string

    group_name = str(row['Grupo']).strip().upper()

    parts = group_name.split('-')

    strain = parts[0] # WKY or SHR

    # Age Mapping: AD -> Aged, JOV -> Young, SEM -> Senescent

    raw_age = parts[-1]

    age_map = {'AD': 'Aged', 'ADULT': 'Aged', 'JOV': 'Young', 'YOUNG': 'Young', 'SEM': 'Senescent'}

    age_label = age_map.get(raw_age, raw_age)

    return pd.Series([strain, age_label])

\# Apply translations

df[['Strain', 'Age']] = df.apply(process_groups_en, axis=1)

\# Calculate Relative Emax

df['Relative Emax'] = (df['Emax'] / df['KCL']) * 100

\# 2. PCA ANALYSIS

features_cols = ['pD2', 'Emax', 'KCL', 'Relative Emax']

df_clean = df.dropna(subset=features_cols).reset_index(drop=True)

x_scaled = StandardScaler().fit_transform(df_clean[features_cols])

pca = PCA(n_components=2)

components = pca.fit_transform(x_scaled)

df_pca = pd.DataFrame(data=components, columns=['PC1', 'PC2'])

df_pca = pd.concat([df_pca, df_clean[['Strain', 'Age', 'Substance']]], axis=1)

\# 3. GENERATE PLOTS (WKY vs SHR)

fig, axes = plt.subplots(1, 2, figsize=(18, 8), sharex=True, sharey=True)

strains = ['WKY', 'SHR']

palette = {'PE': '#1f77b4', 'CORT': '#ff7f0e', 'BMY': '#2ca02c', '5MU': '#d62728'}

markers = {'Young': 'X', 'Aged': 'o', 'Senescent': 's'}

for i, strain in enumerate(strains):

    subset = df_pca[df_pca['Strain'] == strain]

    ax = axes[i]

    if not subset.empty:

        sns.scatterplot(ax=ax, data=subset, x='PC1', y='PC2',

                        hue='Substance', style='Age',

                        hue_order=['PE', 'BMY', '5MU', 'CORT'],

                        style_order=['Young', 'Aged', 'Senescent'],

                        palette=palette, markers=markers, s=250, alpha=0.8, edgecolor='w')

    # Draw Loading Vectors

    loadings = pca.components_.T * 2.5

    for j, feat in enumerate(features_cols):

        ax.arrow(0, 0, loadings[j, 0], loadings[j, 1], color='red', alpha=0.2, head_width=0.1)

        ax.text(loadings[j, 0]*1.2, loadings[j, 1]*1.2, feat,

                color='red', fontsize=12, fontweight='bold', ha='center')

    ax.set_title(f'{strain} Rats - Vascular Profile', fontsize=18, fontweight='bold')

    ax.set_xlabel('PC1 (Efficacy / Contractile Strength)', fontsize=13)

    ax.set_ylabel('PC2 (Sensitivity / Aging Profile)', fontsize=13)

    ax.grid(True, linestyle=':', alpha=0.5)

    ax.axhline(0, color='black', lw=1, alpha=0.1)

    ax.axvline(0, color='black', lw=1, alpha=0.1)

    ax.legend(title='Legend', loc='upper right')

plt.tight_layout()

plt.show()

print(f"Total Explained Variance: {pca.explained_variance_ratio_.sum():.2%}")
