# Supplementary material for: Corticosterone binds α1A-adrenoceptors with high affinity and induces strain-dependent vascular desensitization in female spontaneously hypertensive rats
Source: Hypertens Res. 2026 Aug 6;49(10):2803–16. doi: 10.1038/s41440-026-02760-z (PMC13634920; doi:10.1038/s41440-026-02760-z)
Supplement: Supplementary file 3 [file 41440_2026_2760_MOESM3_ESM.docx]

**Corticosterone binds α1A-adrenoceptors with high affinity and induces strain-dependent vascular desensitization in female spontaneously hypertensive rats**

Yanina A. Santander-Plantamura^a**^, Karen Nuñez-Solano^b^, Santiago C. Sigrist-Flores^a,c^, Itzell A. Gallardo-Ortíz^a^, Enrique Hong^d^, Rafael Villalobos-Molina^a*^

a. Unidad de Investigación en Biomedicina, Facultad de Estudios Superiores Iztacala, Universidad Nacional Autónoma de México, Tlalnepantla, Edo. de México, México.
b. Carrera de Químico Farmacéutico Biólogo, Facultad de Estudios Superiores Zaragoza, Universidad Nacional Autónoma de México, Iztapalapa, Ciudad de México, México.
c. Carrera de Médico Cirujano, Facultad de Estudios Superiores Iztacala, Universidad Nacional Autónoma de México, Tlalnepantla, Edo. de México, México.
d. Departamento de Farmacobiología, Centro de Investigación y de Estudios Avanzados, Sede Sur, Ciudad de México, México.

* correspondence author: Rafael Villalobos-Molina

** co-correspondence author: Yanina A. Santander-Plantamura

Unidad de Investigación en Biomedicina, Facultad de Estudios Superiores Iztacala Universidad Nacional Autónoma de México,

Ave. de Los Barrios 1, Los Reyes Ixtacala, Tlalnepantla, 54090. Estado de México, México

Tel. +52 5623-1333 ext 39795

Email: [villalobos@unam.mx](mailto:villalobos@unam.mx); [santander.plantamura@gmail.com](mailto:santander.plantamura@gmail.com);

**Supplementary data 3 – Docking parameters**

**Supplementary Figure 1:** **Protein-protein interaction network of α1-adrenoceptor signaling components.**

The protein-protein interaction (PPI) network was generated using the Search Tool for the Retrieval of Interacting Genes/Proteins (STRING) database (version 11.5; [https://string-db.org](https://string-db.org/)) [1]. The network includes the following query proteins: α_1A_-adrenoceptor (Adra1a), α_1D_-adrenoceptor (Adrad), Gαq/11 family members (Gna11, Gna12, Gna13, Gna14, Gna15), L-type calcium channel subunit (Cacna1a), and syntrophins (Sntb1, Sntb2), which are scaffold proteins involved in the localization and clustering of dystrophin-associated protein complexes. The confidence score was set to medium (0.400), and disconnected nodes were hidden. Lines (edges) represent predicted functional associations based on curated databases, experimentally determined interactions, gene co-expression, and text mining. Nodes are color-coded according to their connectivity degree. This network illustrates the close functional relationships between α1-adrenoceptors, G proteins, calcium channels, and downstream signaling effectors relevant to vascular smooth muscle contraction.

**Supplementary Methods: Homology modeling of the α_1D_-adrenoceptor**

The three-dimensional structure of the rat α_1D_-adrenoceptor was generated by homology modeling using the Robetta full-chain protein structure prediction server ([2]; [https://robetta.bakerlab.org](https://robetta.bakerlab.org/)). The amino acid sequence of the rat α_1D_-adrenoceptor (UniProt ID: P23944) was submitted as a FASTA query. The Robetta server identified suitable structural templates, performed sequence alignment, and generated the model using the RosettaCM comparative modeling protocol, which combines template-based modeling with ab initio fragment assembly for regions lacking homology .

The resulting model was validated by superimposition onto the cryo-electron microscopy structure of the human α_1A_-adrenoceptor (PDB ID: 7YMH) using UCSF Chimera [3]. The root-mean-square deviation (RMSD) between the two structures was 0.732 Å when calculated over 255 pruned atom pairs, indicating a high degree of structural similarity. Considering all 270 aligned atom pairs, the RMSD was 2.780 Å, which remains well within the acceptable range for homology models.

**Supplementary Results: Model validation of the α_1D_-adrenoceptor homology structure**

The quality of the homology model for the rat α_1D_-adrenoceptor was assessed using MolProbity [4, 5], a comprehensive structure-validation web server. The Ramachandran plot analysis revealed no outliers, with 96.78% of residues located in favored regions (Figure 2). This percentage is slightly below the 98% goal, which is acceptable for a homology model of a GPCR, given the inherent flexibility of these transmembrane proteins. No poor rotamers were detected (0.00%), and the rotamer favorable score reached 100.00%, well above the 98% threshold. The Rama distribution Z-score was -0.61 ± 0.35, well within the acceptable range (|Z| < 2), indicating that the backbone dihedral angles are consistent with those expected for a well-folded protein. Only one Cβ deviation greater than 0.25 Å was observed (0.20%), representing a single outlier. Regarding covalent geometry, bad bonds accounted for 0.12% of all bonds, while bad angles represented 0.22% of all angles, values that are typical for computationally generated models. A total of 5,806 angles were evaluated. No cis prolines were detected, although one cis non-proline (0.19%) and two twisted peptides (0.36%) were identified, which are within acceptable limits for a homology model. The CaBLAM outlier rate was 3.8%, slightly above the 1.0% goal, likely reflecting the complexity of modeling GPCR loop regions. The CA geometry outlier rate was 2.15%. Additionally, the overall model quality was assessed using the ERRAT server [6], which evaluates non-bonded atomic interactions. The overall quality factor was 97.44%, indicating a highly reliable model with excellent atomic packing and interaction statistics. No chiral volume outliers were detected.

Taken together, these validation metrics confirm that the homology model of the rat α_1D_-adrenoceptor is of high quality and suitable for molecular docking studies.

**Supplementary Figure 2: Molprobity validation of the α_1D_-adrenoceptor homology model. Ramachandran plot showing the distribution of backbone dihedral angles (phi/psi).** Favored regions are colored in dark green, allowed regions in light green. No outliers were detected. The ERRAT plot (inset) displays an overall quality factor of 97.44%, indicating good non-bonded atomic interactions.

**Detailed molecular docking protocol**

**Ligand and receptor preparation**

Corticosterone was prepared using UCSF Chimera [3]. The structure was processed with the Dock Prep tool, which included the addition of hydrogen atoms. The prepared ligand was then saved in MOL2 format for subsequent docking analysis. The α_1A_-adrenoceptor structure was also prepared using UCSF Chimera. Non-relevant chains were removed, and all water molecules were deleted to avoid interference with the docking process. The cleaned receptor structure was then saved in PDB format for further analysis.

**α_1A_-adrenoceptor crystal structure and α_1D_-adrenoceptor homology model**

The crystal structure of the α_1A_-adrenoceptor was obtained from the Protein Data Bank (PDB code: 7YMH; [7]). The grid center was set at x = 118.858, y = 121.960, z = 143.227 with a grid box of 40 × 40 × 40 Å and 0.375 Å spacing. The homology-model grid center was set at x = 129.636, y = 121.94, z = 139.103, with a grid box of 40 × 40 × 40 Å and 0.375 Å spacing.

**Docking protocol and validation**

The docking methodology was validated by redocking the co-crystallized ligand into the α_1A_-adrenoceptor crystal structure (PDB ID: 7YMH) using AutoDock Vina (RRID:SCR_011958; [8]). The root-mean-square deviation (RMSD) between the redocked pose and the crystallographic ligand conformation was calculated using the alignment tool in PyMOL (The PyMOL Molecular Graphics System, Version 2.0; Schrödinger, LLC). The calculated RMSD was 0.60 Å (over 9 aligned atoms; 1 outlier atom rejected, final alignment over 8 atoms with RMSD = 0.00 Å), confirming the reliability of the docking protocol for subsequent virtual screening and binding mode analysis [9, 10].

A hybrid Lamarckian Genetic Algorithm was employed, using an initial population of 200 randomly placed individuals. Kollman partial charges were assigned to all protein atoms, while Gasteiger partial charges were assigned to the ligands. All preparations and parameterizations were performed using AutoDock Tools 1.5.6 (RRID:SCR_012746; [11]) AutoDock4 in a Linux operating system (Fedora 22) was used for molecular docking. Finally, the Gibbs free energy (ΔG), and the dissociation constant (Ki), the number of interactions, the binding distance, and type of interactions were obtained with Discovery Studio 4.0 software (RRID:SCR_008398).

**Supplementary Table 1: Binding energies and interaction profiles of corticosterone and phenylephrine with α₁_A_- and α₁_D_-Adrenergic Receptors from molecular docking analysis.**

|  | α_1A_-adrenoceptor | α_1D_-adrenoceptor |
| --- | --- | --- |
| Phenylephrine | **Polar:** Asp106 (TM3.32)  **H-bond:** Met292 (TM6.55), Cys110 (TM3.36)  **π–cation:** Phe312 (TM7.39)  **π- π T Shaped:** Phe288 (TM6.51)  **π- Sigma:** Val107 (TM3.33), Tyr316 (TM7.43) | **Polar:** Asp170 (TM3.32)  **π–Sigma:** Val171 (TM3.33)  **H-bond:** Trh243 (ECL2)  **π – alkyl:** Ile242 (ECL2) |
| Corticosterone | **π–alkyl interactions:** Met292 (TM6.55), Tyr184 (TM5.38), and Phe288 (TM6.51), Cys110 (TM3.36), Val107 (TM3.33), Phe289 (TM6.52), Val185 (TM5.38), Trp285 (TM6.48)  **H-bond:** Asn179 (ECL2) | **H-bond:** Trh243 (ECL2)  **π – alkyl:** Ile242 (ECL2), Phe382 (TM7.39), Phe358 (TM6.51), Trp355 (TM6.48), Cys174 (TM3.36), Phe257 (TM5.47), Val171 (TM3.33) |
| Binding Energy | **Phenylephrine: -6.49 Kcal/mol**  **Corticosterone: -9.14 Kcal/mol** | **Phenylephrine: -6.28 Kcal/mol**  **Corticosterone: -7.49 Kcal/mol** |
| Ki | **Phenylephrine: 17.36 µM**  **Corticosterone: 198.13 nM** | **Phenylephrine: 24.88 µM**  **Corticosterone: 3.25 µM** |

**Reference**

[1]	Szklarczyk D, Nastou K, Koutrouli M, et al. The STRING database in 2025: protein networks with directionality of regulation. *Nucleic Acids Res* 2025; 53: D730–D737.

[2]	Kim DE, Chivian D, Baker D. Protein structure prediction and analysis using the Robetta server. *Nucleic Acids Res*; 32. Epub ahead of print July 1, 2004. DOI: 10.1093/nar/gkh468.

[3]	Pettersen EF, Goddard TD, Huang CC, et al. UCSF Chimera - A visualization system for exploratory research and analysis. *J Comput Chem* 2004; 25: 1605–1612.

[4]	Chen VB, Arendall WB, Headd JJ, et al. MolProbity: All-atom structure validation for macromolecular crystallography. *Acta Crystallogr D Biol Crystallogr* 2010; 66: 12–21.

[5]	Williams CJ, Headd JJ, Moriarty NW, et al. MolProbity: More and better reference data for improved all-atom structure validation. *Protein Science* 2018; 27: 293–315.

[6]	Colovos C, Yeates TO. Verification of protein structures: Patterns of nonbonded atomic interactions. *Protein Science* 1993; 2: 1511–1519.

[7]	Toyoda Y, Zhu A, Kong F, et al. Structural basis of α1A-adrenergic receptor activation and recognition by an extracellular nanobody. *Nat Commun*; 14. Epub ahead of print December 1, 2023. DOI: 10.1038/s41467-023-39310-x.

[8]	Trott O, Olson AJ. AutoDock Vina: Improving the speed and accuracy of docking with a new scoring function, efficient optimization, and multithreading. *J Comput Chem* 2010; 31: 455–461.

[9]	Boittier ED, Tang YY, Buckley ME, et al. Assessing molecular docking tools to guide targeted drug discovery of cd38 inhibitors. *Int J Mol Sci* 2020; 21: 1–19.

[10]	Yusuf D, Davis AM, Kleywegt GJ, et al. An alternative method for the evaluation of docking performance: RSR vs RMSD. *J Chem Inf Model* 2008; 48: 1411–1422.

[11]	Morris GM, Ruth H, Lindstrom W, et al. Software news and updates AutoDock4 and AutoDockTools4: Automated docking with selective receptor flexibility. *J Comput Chem* 2009; 30: 2785–2791.
